# Mapping vocal health risk for active vocal users across European urban soundscapes: a computational framework with exploratory empirical evaluation

**DOI:** 10.3389/fpubh.2026.1933020

**Published:** 2026-09-03

**Authors:** Yuwen Sun

**Affiliations:** College of Design and Innovation, Tongji University, Shanghai, China

**Keywords:** active vocal users, Lombard effect, subglottal pressure, urban soundscape, vocal health risk

## Abstract

Urban soundscape assessments focus on the listener—the person who hears an environment—while largely overlooking the person who must produce sound within it. However, speaking or singing in noisy settings imposes substantial physiological demands due to Lombard effect, which causes vocal intensity to increase as ambient noise raises. In this study, we present a computational framework that integrates the Lombard effect, subglottal pressure estimation, and signal-to-noise ratio (SNR) to translate urban noise levels into quantitative estimates of vocal load. Calibrated against professional-singer data, the subglottal-pressure estimates reproduce independently measured sound levels to within 0.7 dB. This framework has been applied to strategic road-traffic noise reported under the European Environmental Noise Directive, mapping vocal health risks across urban areas in Denmark, the Netherlands, and France. In these regions, high-risk zones account for 4.3–5.6% of the noise-affected area; an exploratory analysis of 16 singers recorded in two environments is directionally consistent with the framework’s central assumption. The framework’s principal finding is a divergence between its two risk indicators. As vocal compensation strengthens, SNR-based risk falls, while estimated subglottal pressure rises. This means that successfully compensating for sound typically raises audibility and adds to the physiological load, so an assessment based only on SNR indicates improvement at the moment the hazard increases. Communication-based metrics can therefore conceal, rather than reveal, rising vocal load, and should not be used in isolation. More broadly, the results argue for extending the human-centered soundscape paradigm to those who actively produce sound in the city, rather than only to those who receive it.

## 1 Introduction

The acoustic environment of cities has become an increasingly prominent concern in urban design scholarship, with growing attention to how sound and auditory experience can be integrated into the planning, design, and management of public spaces (1). Rising noise from traffic, construction, and dense human activity is now a recognized threat to public health. Epidemiological evidence links transportation noise to increased cardiovascular morbidity and mortality, with the strongest evidence for ischemic heart disease (2); night-time road traffic noise is associated with disturbed sleep, including difficulty falling asleep and waking too early (3), alongside broader concerns about noise-related cognitive impairment (4).

Against this largely hazard-oriented account, soundscape research, formalized through International Organization for Standardization (ISO) 12913 (5), has advanced a more nuanced understanding of how people perceive and respond to their acoustic surroundings. Across indoor and outdoor settings, the field has increasingly adopted human-centered approaches that view sound not merely as a hazard to be minimized, but as a resource to be shaped, emphasizing the qualitative character of the acoustic environment rather than sound level alone (6). Accordingly, its attention has concentrated on perceptual outcomes: appraisal, pleasantness, and wellbeing (7, 8).

This is a genuine advance, but it shares a vantage point with the hazard tradition it refines. Both regard the individual as someone to whom sound happens, a listener whose experience an environment either harms or improves. Neither has much to say about the individual who acts within the acoustic environment by producing sound, and whose body is taxed not by hearing the noise but by having to be heard over it. The physiological consequences of urban acoustic environments for those who actively produce sound within them have, in short, remained largely unexplored.

The individuals most directly exposed to this gap are those whose work or activity depends on sustained vocal production. Professional voice users, including singers, actors, and other performers, form one visible population for whom the voice is constitutive of their work (9), and for whom environmental conditions are established contributors to occupational voice disorders (10). Their demands can be considerable: among contemporary commercial gig singers, Australian professionals surveyed reported performing for hours at a time, several times a week, frequently pushing vocal output to stay audible over continuous background noise in venues without acoustic treatment (11). Among outdoor voice users, street performers, market vendors, outdoor educators, and tour guides face the same basic predicament, raising vocal effort to compete acoustically with their surroundings, a pattern that contributes to vocal fatigue and impairment over time (12).

Recognized occupational groups, however, represent only the visible tip of a much larger population. Choir members, street performers, tour guides, and community musicians routinely sustain vocal output in open-air venues, public squares, and roadside rehearsal spaces, yet fall outside the occupational categories through which vocal risk is typically studied. Regardless of classification, the acoustic conditions of these environments directly shape vocal production and impose physiological demands. Accordingly, the present study focuses on outdoor urban vocal activities, where environmental road-traffic exposure can be characterized using European Environmental Noise Directive (END) data, rather than on indoor occupational settings that lie beyond the coverage of those data.

Occupational and clinical voice science has, in parallel, mapped what sustained effort under noise does to the voice. Prolonged vocal effort in noisy conditions is associated with increased vocal loading (13) and elevated phonotraumatic risk (14, 15); the mechanism by which noise drives effort is itself well characterized. As ambient noise rises, speakers and singers involuntarily raise their output, known as the Lombard effect, and compensation sustained in this way causes physiological load to accumulate over time (16, 17). Yet this body of knowledge has stayed largely within occupational and clinical settings. It has rarely entered urban soundscape research, which remains centered on the listener, and the built-environment literature has not taken up the vocal-health consequences of the acoustic decisions it makes. Two fields that describe the same physical situation—a voice working against ambient noise—have thus developed in near isolation, leaving a specific gap: no method translates an urban noise level into a physiologically meaningful estimate of vocal load, and vocal health has never been operationalized as a dimension of soundscape assessment.

This study builds that bridge. It develops an interdisciplinary computational framework that couples three speech-science components—the Lombard effect (18), subglottal pressure estimation (19, 20), and signal-to-noise ratio (SNR)—into a single pipeline that estimates vocal health risk from urban noise exposure. Here, vocal health risk refers specifically to the physiological vocal load that ambient noise imposes on active vocal users. It does not cover clinical voice health in full, which also depends on factors such as phonation duration, hydration, technique, medical history, and individual susceptibility beyond the model’s scope. The pipeline is calibrated against representative noise scenarios using empirical data from professional singers (21, 22), and its subglottal-pressure estimates are calibrated against independently measured sound levels in classical male singers, which they reproduce to within 0.7 dB. Applied to open noise-exposure data reported under the European Environmental Noise Directive, the framework generates city-scale vocal health risk maps for several European agglomerations. Its central behavioral assumption—that vocal output increases with ambient noise—is independently evaluated through acoustic analysis of 16 student singers recorded in two acoustically distinct environments.

The present application is limited to outdoor urban vocal activity because the framework relies on outdoor road-traffic noise data. This framework extends the human-centered soundscape paradigm beyond listeners to those who must actively produce sound within the city’s acoustic environment.

## 2 Theoretical background

### 2.1 The Lombard effect in singers

The Lombard effect, first described by Étienne Lombard (23), is among the most robust findings in voice science, observed across species, tasks, and noise conditions (24). In singers, it is not fixed but shaped by training. Bottalico et al. (16) found a Lombard slope of 0.13 dB/dB in classically trained professionals, well below the 0.21 dB/dB observed in non-professionals, meaning that the trained voice pushes back less against rising noise. The reason is a shift in what the singer listens to: training moves control away from external auditory feedback toward internal, kinesthetic cues, so that trained singers maintain pitch under auditory masking far better than instrumentalists or non-musicians (25). A suppressed Lombard slope is thus not a smaller reflex but a trained substitution of one feedback channel for another.

This training-dependence has a direct consequence for how the present study should be read. The singer sound pressure level (SPL) values on which it builds, 78.9–81.9 dBA at 1 m across accompaniment levels of 70–90 dBA (16), come from professionals, the group that compensates least. They were also obtained under stable laboratory accompaniment, whereas the noise of real urban environments fluctuates and typically reaches higher levels, ranging from 51.8–65.5 dB(A) in recreational areas (26) to 85–90 dB(A) on high-traffic streets (27). Both facts point in the same direction: a less-trained voice in a noisier, less predictable environment would compensate more, not less. The estimates that follow should therefore be read as a floor—the load under the most favorable assumptions—rather than a typical value.

### 2.2 Subglottal pressure as the primary vocal load indicator

Subglottal pressure ( $P_{s}$ ), the aerodynamic driving force beneath the vocal folds during phonation, is the physiological variable that most directly governs vocal loudness. The link between the two is quantitative and well characterized: Sundberg et al. (20) found that a doubling of $P_{s}$ raises SPL by approximately 10 dB in professional male singers, a value close to the 8–9 dB predicted by acoustic theory. This relationship underpins the present framework, allowing the required acoustic output to be translated into the underlying biomechanical demand needed to produce it.

That demand has a well-mapped range in trained voices. Cleveland et al. (22) measured $P_{s}$ in professional country singers, finding values mostly below 45 $\mathrm{cm}\;H_{2}O$ in performance, with peaks near 59 $\mathrm{cm}\;H_{2}O$ ; Björkner et al. (21) showed that classically trained baritones hold a consistent phonation mode across a wide $P_{s}$ range, raising pressure chiefly to meet the demands of higher fundamental frequencies rather than changing how they phonate. $P_{s}$ , in other words, is both the lever of loudness and a stable, interpretable index of what phonation is costing the singer.

That cost is not merely metabolic; it is borne by tissue. Elevated $P_{s}$ raises vocal-fold collision stress, and sustained high pressure is an established antecedent of nodules, contact ulcers, and chronic dysphonia in the clinical literature (10, 14, 28). The vocal dose framework by Titze et al. makes the accumulation explicit (29): cumulative tissue-vibration exposure is a function of both phonation duration and loudness, so raising output not only increases the immediate cost but also contributes to a dose that builds over time. For a vocalist maintaining elevated output throughout a noisy rehearsal or performance, each additional decibel is not a fixed price but an accruing one, shortening the margin before vocal load progresses to injury (29, 30).

### 2.3 Vocal health as a missing dimension of human-centered soundscape

The soundscape paradigm formalized in ISO 12913 (5) already treats the acoustic environment as more than a physical sound level: it is a perceptual and contextual construct, meaningful only through the person experiencing it. This is the same human-centered turn that reframed noise from a hazard to be minimized to a quality to be shaped (6). Its instruments, however, remain calibrated to reception. Even the most advanced of them optimize for the listener: automatic masker-selection systems, for instance, can now predict and improve the ISO Pleasantness of a noisy environment without lowering its physical level at all (31), an achievement defined entirely in terms of how sound is perceived. Vocal health enters this paradigm not as a competing metric but as its missing half: a dimension that operates on physiological demand rather than perceptual experience, and that can be assessed alongside pleasantness rather than in place of it. The framework developed below is an attempt to supply that dimension.

## 3 Methods

### 3.1 Scenario definition

**Figure 1:**
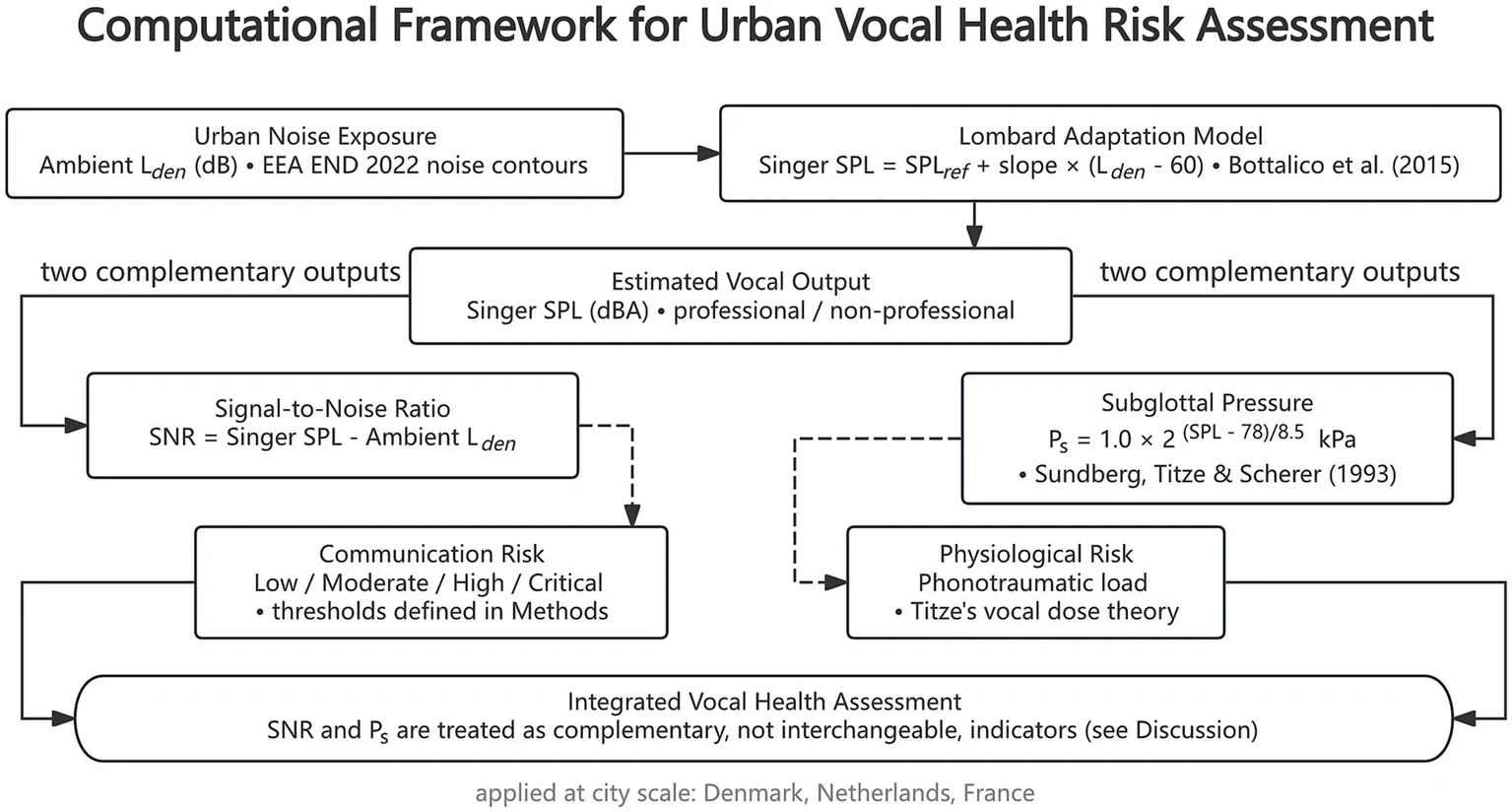
Computational framework for urban vocal health risk assessment. The pipeline integrates the Lombard adaptation model, subglottal pressure (*P*s) estimation, and signal-to-noise ratio (*SNR*) into two complementary risk indicators, communication risk (*SNR*-based) and physiological risk ( $P_{s}$ -based). SNR-based risk thresholds are defined in the Methods. The two indicators are treated as complementary rather than interchangeable.

Three representative urban acoustic scenarios were defined based on documented real-world noise measurements and World Health Organization (WHO) guidelines. Scenario A (~60 dB) represents a rehearsal or performance space where urban noise is only partially attenuated rather than an acoustically isolated studio, a level consistent with the U. S. Environmental Protection Agency (EPA) outdoor levels for urban residential environments (32) and at the lower boundary of the range active vocal users encounter. Scenario B (Urban Street, ~75 dB) is grounded in empirical street-level measurements: a study across 99 New York City street sites found a mean noise level of 73.4 dBA with a range extending to 95.0 dBA (27). Scenario C (Noisy Plaza/High-Traffic Area, ~85–90 dB) represents busy commercial or arterial road conditions, within the documented upper range of urban street noise and above the WHO-recommended ceiling for road traffic noise exposure (4) (Figure 1).

For each scenario, the required professional singer vocal output was estimated from Bottalico et al. (16) Table II, using Set 1 (standard acoustic condition), Group 1 (professional singers) data. The singer SPL values of 78.9, 80.0, and 81.9 dBA correspond, respectively, to accompaniment levels of 70, 80, and 90 dBA, serving as proxies for the ambient noise conditions in Scenarios A, B, and C. This is an approximation: laboratory accompaniment level and field ambient noise are not equivalent quantities, and the mapping is intended to anchor the scenarios in measured singer behavior rather than to assert their acoustic identity.

### 3.2 Subglottal pressure estimation

Subglottal pressure was estimated using the quantitative relationship established by Sundberg et al. (20), who reported that a doubling of $P_{s}$ raises SPL by approximately 10 dB in professional male singers, consistent with the 8–9 dB predicted by acoustic theory. A conservative value of 8.5 dB per doubling was adopted here, which yields higher estimated $P_{s}$ for a given SPL and therefore does not overstate vocal load. At a reference $P_{s}$ of 1.0 kPa, the measured SPL was 84 dB at 0.5 m (20), corresponding to approximately 78 dB at 1 m after a 6 dB inverse-square-law adjustment. Solving for $P_{s}$ from a target SPL:


$$P_{s}(\mathrm{kPa})=1.0\times 2^{({\mathrm{SPL}}_{\text{target}}-78)/8.5}$$

Converting to ${\mathrm{cmH}}_{2}O$ ( $1\;\mathrm{kPa}=10.197\;{\mathrm{cmH}}_{2}O$ ) yields physiologically interpretable values directly comparable to the literature. All calculations were implemented in Python 3.13. Vocal tract configuration was not varied across scenarios, as the framework targets aerodynamic vocal load estimation rather than articulatory adaptation; future extensions should incorporate Lombard-driven changes in formant tuning and spectral tilt.

### 3.3 Framework calibration against independently measured subglottal pressure

To verify that the estimation procedure described in Section 3.2 reproduces the empirical pressure–intensity relationship observed in classical male singing, rather than simply reflecting its underlying assumptions, we compared its predictions with subglottal pressures and sound levels measured directly in professional male singers by Sundberg et al. (20). In that study, a mean SPL of 84 dB (standard deviation [SD] = 6) at 0.5 m corresponded to a reference $P_{s}$ of 1.0 kPa across 10 singers, and a doubling of $P_{s}$ raised SPL by approximately 10 dB. Over the range of subglottal pressure, the framework estimates for urban singing (11.0–14.0 ${\mathrm{cmH}}_{2}O$ across scenarios; 13.1 ${\mathrm{cmH}}_{2}O$ maximum at city scale), the predicted SPL values fell within 0.7 dB of the corresponding measured levels, well inside the 6 dB inter-singer standard deviation reported by Sundberg et al. (20). The estimated pressures themselves occupy the soft-to-moderate portion (roughly the lower quarter) of the subglottal pressure ranges independently measured in operatic baritones (approximately 5–40 ${\mathrm{cmH}}_{2}O$ ; 21) and country singers (5–59 ${\mathrm{cmH}}_{2}O$ ; 22). Two features of this comparison should be noted. First, the estimation formula is anchored to the same pressure-intensity study used for comparison (20), so the agreement in the rate of SPL gain per pressure doubling is partly structural; the more informative test is the absolute-level agreement at the reference pressure, which draws on the measured 0.5 m SPL value not otherwise used to construct the model. Second, this reference material derives from classically trained male voices, and singing styles that employ greater glottal adduction produce lower SPL at a given $P_{s}\;$ (22); the estimator is therefore calibrated for classical phonation, and its extension to higher-adduction or untrained vocal styles would require style-specific recalibration (Section 5.3).

### 3.4 City-scale empirical application

To extend the framework beyond hypothetical scenarios, we applied it to open noise-exposure data from the 2022 reporting round of the EU Environmental Noise Directive (Directive 2002/49/EC) (33). Road-traffic noise contour data for urban agglomerations were retrieved from the European Environment Agency (EEA) Strategic Noise Mapping dataset in GeoPackage format, released under the Open Database License (ODbL) and freely accessible via the EEA Spatial Data Infrastructure (34). Data were obtained for three countries representing different urban morphologies and regulatory contexts: Denmark, the Netherlands, and France. The analysis was restricted to the layer NoiseContours_roadsInAgglomeration_Lden, which contains noise contour zones for road traffic within urban agglomerations, expressed as day-evening-night equivalent sound level (Lden).

Each noise contour zone is assigned a categorical noise band label (e.g., Lden5559, Lden6064, LdenGreaterThan75). The lower boundary of each band was extracted via regular expression parsing of the category string, and a representative midpoint value was assigned to each band (57, 62, 67, 72, and 77 dB for the 55–59, 60–64, 65–69, 70–74, and ≥75 dB bands, respectively). The midpoint of the highest open-ended band (≥75 dB) was conservatively set to 77 dB, consistent with the lower boundary of that category. For each noise contour zone, the ambient Lden midpoint value was used as input to the vocal load pipeline described in Sections 3.1–3.2.

Lden is a long-term, annually averaged descriptor that applies evening and night penalties (+5 and +10 dB). It is therefore not equivalent to the instantaneous accompaniment level from which the Lombard slope was derived, nor to the sound level a vocalist experiences at any single moment. The pipeline therefore operates at the level of chronic relative exposure and is based on a simple assumption: locations with higher annual Lden expose vocalists to systematically higher instantaneous noise over time, and hence to higher long-term compensatory load. The estimated Singer SPL and $P_{s}$ are accordingly interpreted as relative indicators of chronic compensatory load across locations, and the SNR as a relative, long-term exposure index for comparing zones and countries rather than an instantaneous physical measurement. Because vocal load accumulates over time, a long-term averaged descriptor is an appropriate scale for such an indicator, even if it is not the right scale for any single phonation event.

Singer SPL was estimated using the Lombard adaptation model:


$$\text{Singer}\;\mathrm{SPL}\;(\mathrm{dBA})=78.9+0.13\times (L_{\mathrm{den}}-60)$$

Where 78.9 dBA is the measured output of professional singers at the lowest tested accompaniment level (70 dBA); it is used here as the baseline for a Lombard extrapolation anchored at 60 dBA (16), and 0.13 dB/dB is the Lombard slope for classically trained professional singers. Subglottal pressure was then estimated from Singer SPL using the formula in Section 3.2, and SNR was computed as:


$$\mathrm{SNR}\;(\mathrm{dB})=\text{Singer}\;\mathrm{SPL}-L_{\mathrm{den}}$$

Each noise contour zone was assigned a vocal health risk level based on its estimated SNR, classified as Critical (SNR < 0 dB), High (0 ≤ SNR < +5 dB), Moderate (+5 ≤ SNR < +10 dB), or Low (SNR ≥ +10 dB). These SNR bands are operational definitions adopted for this study rather than established clinical cut-offs. The 0 dB boundary marks the point at which the singer’s own output is masked by ambient noise, while the +5 and +10 dB boundaries are pragmatic strata used to describe the relative distribution of exposure rather than to diagnose individual risk. Spatial analysis was performed in Python 3.13 using GeoPandas (version 0.14), with geometries reprojected to European Petroleum Survey Group (EPSG) code 3035 (European Terrestrial Reference System 1989–Lambert azimuthal equal-area [ETRS89-LAEA]) for area-accurate calculations. The proportion of total noise-affected area falling within each risk category was computed for each country, along with the maximum estimated $P_{s}$ and minimum estimated SNR across all noise zones. Results are reported as area-weighted risk distributions rather than zone counts to account for differences in data granularity across national datasets.

### 3.5 Sensitivity analysis

The Lombard slope used in the primary analysis (0.13 dB/dB) represents the empirically measured value for classically trained professional singers (16). However, the broader population of active vocal users encompasses individuals with a range of vocal training levels, for whom the Lombard slope may differ substantially. Bottalico et al. (16) reported Lombard slopes of 0.21 dB/dB for non-professional singers, and 0.08 and 0.17 dB/dB for professional and non-professional singers, respectively, under restored external auditory feedback. To evaluate the robustness of the city-scale risk estimates, we performed a sensitivity analysis by varying the Lombard slope from 0.10 to 0.25 dB/dB in increments of 0.03–0.05 dB/dB, spanning and slightly extending the empirically reported range.

For each slope value, Singer SPL, $P_{s}$ , and SNR were recomputed for every noise contour zone across all three countries, and the proportion of High-risk area was recalculated. The analysis examined two questions. The first was whether the rank ordering of countries by High-risk area proportion remained stable across the slope range. Because the countries differed by less than 1.5 percentage points in High-risk area, this ordering was examined for stability rather than treated as a substantive cross-national contrast. The second was whether the SNR-based and $P_{s}$ -based risk indicators diverged systematically as the slope increased, motivated by the expectation that higher Lombard slopes, while improving apparent SNR through stronger vocal compensation, simultaneously impose greater subglottal pressure, thereby creating a discrepancy between communication-based and physiological risk assessment.

### 3.6 Exploratory empirical evaluation

This section reports an opportunistic pilot analysis using archival pedagogical recordings. It is intended solely as a directional consistency check on the framework’s core behavioral assumption, not as a formal test of the Lombard model. Sixteen student singers (S01–S08 per environment) performed the opening 12 bars of Caccini’s Amarilli, mia bella under structured teaching conditions, with recordings made in two locations: an instrument storage warehouse (noisier environment) and a large piano rehearsal room located adjacent to a distant construction site (quieter environment). The recordings were originally collected for pedagogical and voice-training purposes rather than for Lombard-effect research. Consequently, the dataset should be regarded as an opportunistic sample for exploratory analysis rather than a purpose-designed experimental dataset. All recordings were made using the same microphone and recording parameters in Waveform Audio File Format (WAV) with consistent gain settings. Each singer produced two takes of the same passage; both takes were retained and averaged for each singer to reduce within-session variability. Singers were assigned to environments as part of an existing teaching arrangement and were not randomly allocated; the two groups therefore constituted independent samples rather than a within-subject comparison.

Environmental noise floor was estimated from the first 5 sec of each recording prior to any vocalization, expressed as root-mean-square (RMS) level in decibels relative to full scale (dBFS). Total vocal output was estimated as the RMS level of the isolated singing segment in dBFS, manually extracted from each recording to exclude teaching dialog and between-phrase pauses. Although ambient noise was not measured using a calibrated sound level meter, and the recordings were not originally collected for Lombard-effect research, the dataset provides an opportunity to examine whether singers in a relatively noisier environment exhibit vocal output patterns consistent with the framework’s predictions. All acoustic analyses were implemented in Python 3.13 using the librosa library (version 0.10). Group differences were assessed using the Mann–Whitney U test (one-tailed, testing the hypothesis that warehouse output exceeds piano room output), with effect size reported as r=Z/√N .

## 4 Results

### 4.1 Subglottal pressure across scenarios

**Figure 2:**
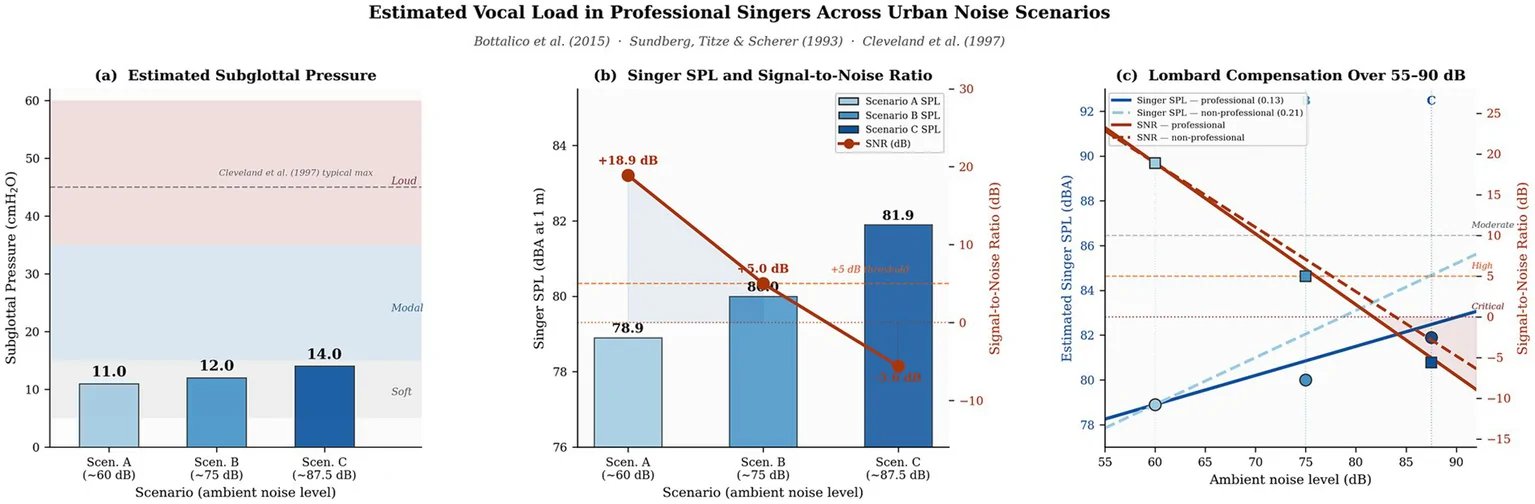
Estimated vocal load in professional singers across three urban noise scenarios. **(A)** Subglottal pressure (P_s_) estimated from singer sound pressure level (SPL) values using the pressure-intensity relationship of Sundberg et al. (20), with literature reference ranges for soft, modal, and loud phonation shown as background bands (22). **(B)** Singer output sound pressure level (SPL) (bars, left axis) and vocal signal-to-noise ratio (SNR; line, right axis) across scenarios; the +5 dB threshold indicates the boundary of the Moderate risk zone. **(C)** Continuous simulation of Singer SPL and SNR across the ambient noise range 55–90 dB, for professional (Lombard slope 0.13 dB/dB) and non-professional (0.21 dB/dB) singers; labeled markers indicate the three calibration scenarios—singer SPL data from Bottalico et al. (16).

Table 1 presents the estimated subglottal pressure for each scenario. Across the three urban noise conditions, $P_{s}$ increases from 11.0 ${\mathrm{cmH}}_{2}O$ in the quiet rehearsal environment to 14.0 ${\mathrm{cmH}}_{2}O$ in the noisy plaza, representing a 27% increase driven by a difference of only 3.0 dBA in required vocal output. While these values fall in the moderate-to-soft phonation range identified by Cleveland et al. (22), they represent the lower bound of a sustained exposure profile: a singer in an 87.5 dB urban environment is not performing a single fortissimo phrase but maintaining this elevated subglottal pressure across an extended rehearsal or performance period (Figure 2).

**Table 1:** Estimated vocal load across three urban noise scenarios.

Scenario	Ambient (dB)	Singer SPL (dBA)	Est. P_s_ cm H_2_O)	SNR (dB)
A: Quiet rehearsal	~60	78.9	11.0	+18.9
B: Urban street	~75	80.0	12.0	+5.0
C: Noisy plaza	~87.5	81.9	14.0	−5.6

SPL, sound pressure level; SNR, signal-to-noise ratio. Singer SPL values were derived from Bottalico et al. (16). P_s_ denotes subglottal pressure and was estimated using Sundberg et al. (20).

### 4.2 The voice-to-noise ratio across scenarios

The SNR column of Table 1 traces how the singer’s audibility margin narrows as noise rises. In Scenario A the margin is comfortable (+18.9 dB); in Scenario B it falls to +5.0 dB, near the level at which auditory self-monitoring begins to degrade; in Scenario C, the most severe modeled condition, it turns negative (−5.6 dB), meaning the singer’s own output is masked by ambient noise. This last case is where both SNR and physiological load become jointly informative: a vocalist who cannot hear their own output tends to push harder, sustaining high subglottal pressure while auditory self-regulation is compromised. It is worth stressing that Scenario C is a deliberately extreme calibration point rather than a typical exposure; whether real urban environments reach it is an empirical question the city-scale analysis directly addresses (Section 4.3), and there the negative-SNR regime does not, in fact, appear. It functions instead as a warning threshold, marking what would occur should ambient noise rise further, rather than describing any zone in the present data.

### 4.3 Cross-city vocal health risk mapping

**Figure 3:**
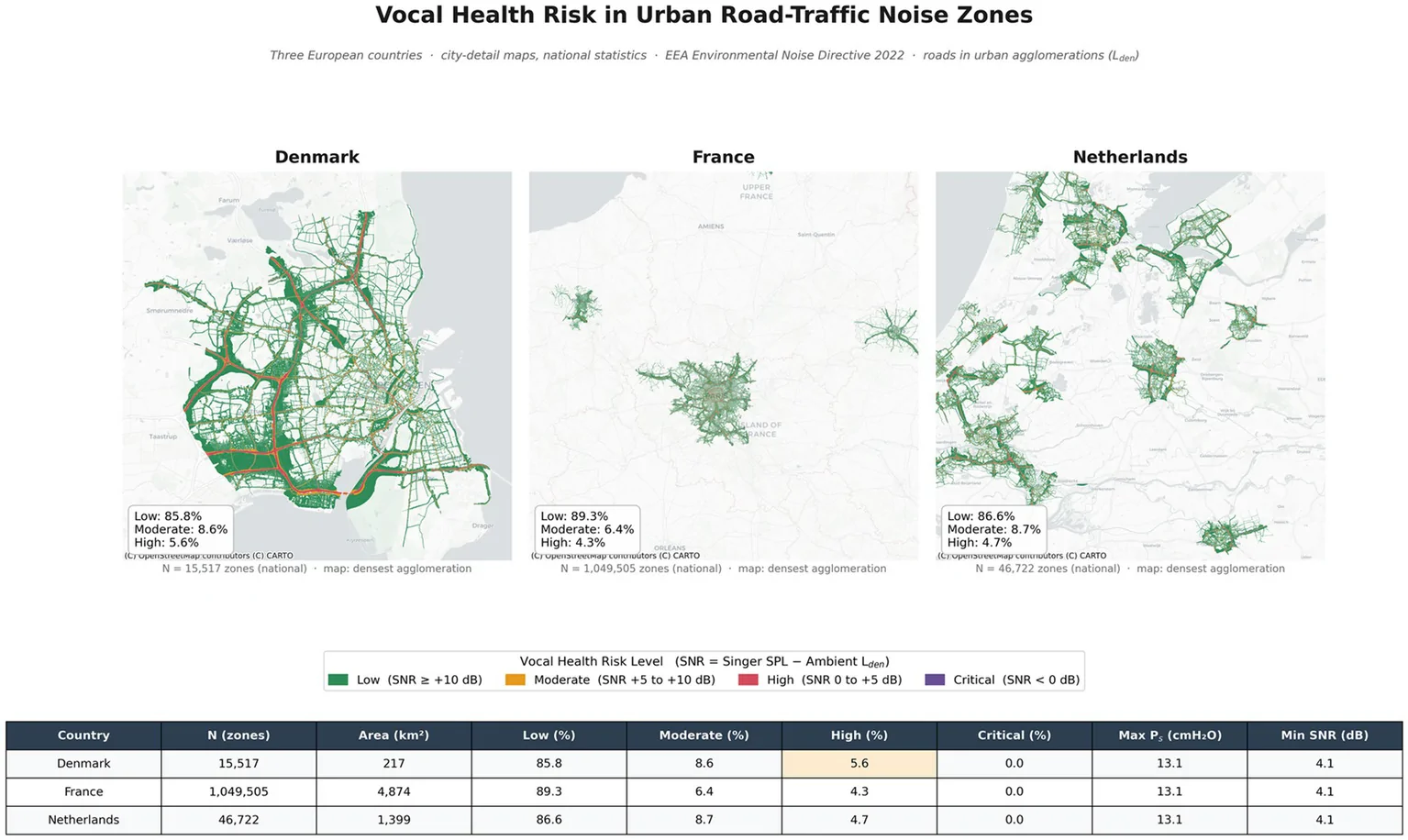
Vocal health risk classification in urban road-traffic noise zones across three European countries [European Environment Agency (EEA), 2022 reporting round, roads in urban agglomerations, day-evening-night equivalent sound level (*Lden*)]. Risk level is determined by the estimated vocal signal-to-noise ratio (*SNR* = Singer *SPL* − Ambient *Lden*), in operationally defined bands: Low (*SNR* ≥ + 10 dB), Moderate (+5 ≤ *SNR* < +10 dB), High (0 ≤ *SNR* < +5 dB), Critical (< 0 dB). Maps show a detail view of the principal urban agglomeration of each country (Copenhagen, Paris, the Randstad) to display risk-zone texture; the zone counts, areas, and risk percentages are national and area-weighted [European Petroleum Survey Group (EPSG) code 3035]. Denmark, the Netherlands, and France show High-risk area proportions of 5.6, 4.7, and 4.3%, respectively. No Critical-risk zones were identified in any country. Vocal load was estimated via Bottalico et al. (16) and Sundberg et al. (20). Map data © OpenStreetMap contributors, licensed under the Open Data Commons Open Database License (ODbL); © CARTO.

Application of the vocal load framework to EEA road traffic noise data across three European countries revealed comparable, modest High-risk proportions (Figure 3; Table 2). High-risk zones occupied a similar share of noise-affected area in Denmark (5.6%), the Netherlands (4.7%), and France (4.3%). Moderate-risk zones were similarly close (Denmark 8.6%, Netherlands 8.7%, France 6.4%), and Low-risk zones dominated in all three (Denmark 85.8%, France 89.3%, and the Netherlands 86.6%).

**Table 2:** Vocal health risk distribution across three European urban agglomerations [European Environment Agency (EEA), 2022 reporting round, road traffic, day-evening-night equivalent sound level (Lden)].

Country	N (zones)	Area (km^2^)	Low (%)	Moderate (%)	High (%)	Critical (%)	Max P_s_ (cm H_2_O)	Min SNR (dB)
Denmark	15,517	217	85.8	8.6	5.6	0.0	13.1	+ 4.1
Netherlands	46,722	1,399	86.6	8.7	4.7	0.0	13.1	+ 4.1
France	1,049,505	4,874	89.3	6.4	4.3	0.0	13.1	+ 4.1

No Critical-risk zones (SNR < 0 dB) were identified in any country. Identical maximum $P_{s}$ and minimum SNR values across countries reflect that all three datasets share the same highest noise band midpoint (77 dB); cross-national differences arise from differences in the spatial distribution of noise bands rather than from the framework parameters. Area proportions are computed using European Petroleum Survey Group (EPSG) code 3035. Maximum subglottal pressure (P_s_) and minimum signal-to-noise ratio (SNR) correspond to the highest noise band (Lden ≥ 75 dB, midpoint 77 dB).

No Critical-risk zones (SNR < 0 dB) were identified in any of the three countries. The minimum estimated SNR across all noise zones in all three countries was +4.1 dB, corresponding to the highest noise band (Lden ≥ 75 dB, represented by a midpoint of 77 dB). This finding indicates that, under the Lombard slope parameterization for professional singers (0.13 dB/dB), European road traffic noise levels regulated in the 2022 END reporting round do not, in aggregate, drive active vocal users into the negative-SNR regime associated with a self-reinforcing Lombard feedback loop. This is consistent with the noise reduction objectives of the END framework, though the broadly similar High-risk proportions across countries suggest this is a general feature of the urban road-noise environment rather than a nationally specific one.

The maximum estimated $P_{s}$ was identical across all three countries (13.1 ${\mathrm{cmH}}_{2}O$ ), because the highest noise band midpoint (77 dB) produces the same vocal load estimate regardless of the country in which it occurs. Cross-country differences in risk distribution therefore arise entirely from differences in the spatial distribution of road-traffic noise levels, rather than from the framework’s physiological parameters. The small residual differences may reflect variations in road network density, traffic patterns, or noise management strategies, although the present dataset does not allow causal attribution.

### 4.4 Sensitivity analysis results

**Figure 4:**
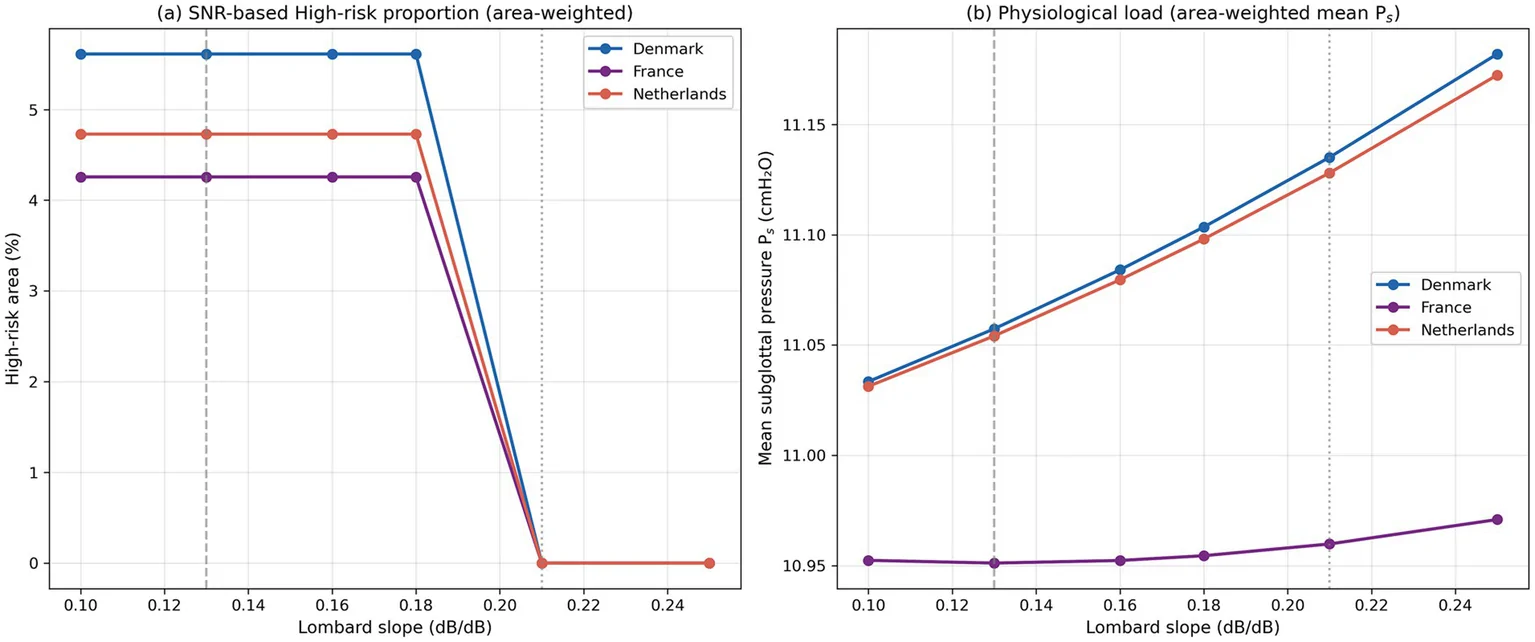
Sensitivity analysis of vocal health risk indicators across the range of Lombard slopes (0.10–0.25 dB/dB). **(a)** Signal-to-noise ratio (SNR)-based high-risk area proportion for each country (area-weighted). Values remain stable at slopes 0.10–0.18 dB/dB (Denmark 5.6%, Netherlands 4.7%, France 4.3%) and fall to 0% at slopes ≥ 0.21 dB/dB as stronger vocal compensation drives SNR above the +5 dB threshold. **(b)** Area-weighted mean subglottal pressure (P_s_). P_s_ increases monotonically with slope for all three countries; Denmark and the Netherlands follow nearly identical trajectories, with France slightly lower throughout. Vertical lines indicate the professional singer slope (0.13 dB/dB, dashed) and non-professional singer slope (0.21 dB/dB, dotted) reported by Bottalico et al. (16). The divergence between panels **(a)** and **(b)** demonstrates that SNR-based and P_s_-based risk indicators can move in opposite directions as compensation strength increases.

Figure 4 presents the sensitivity analysis across Lombard slopes from 0.10 to 0.25 dB/dB. The two panels reveal a systematic divergence between the SNR-based communication risk indicator and the $P_{s}$ -based physiological load indicator as the slope increases.

Panel (a) shows that SNR-based High-risk area proportions were stable across slopes from 0.10 to 0.18 dB/dB (Denmark 5.6%, the Netherlands 4.7%, France 4.3%), with the country ordering preserved throughout this range. At slopes of 0.21 dB/dB and above, High-risk proportions fell to 0% for all three countries. This occurs because stronger vocal compensation drives Singer SPL upward relative to Lden, pushing SNR above the +5 dB High-risk threshold. From the perspective of SNR-based classification alone, higher Lombard slopes therefore appear to eliminate vocal health risk.

Panel (b) shows the opposite pattern. Mean $P_{s}$ increased monotonically with Lombard slope across all three countries, from 10.95–11.03 ${\mathrm{cmH}}_{2}O$ at slope 0.10 to 10.97–11.18 ${\mathrm{cmH}}_{2}O$ at slope 0.25. Denmark and the Netherlands showed nearly identical $P_{s}$ trajectories and the steepest increase, while France, with a larger share of lower-noise zones, remained slightly below them across the range. The ordering by mean $P_{s}$ (France < Netherlands ≈ Denmark) was stable across the entire slope range.

This divergence between the SNR-based and $P_{s}$ -based indicators is the principal observation of the sensitivity analysis, with implications for risk indicator design developed below.

### 4.5 Exploratory empirical evaluation results

Table 3 presents the per-singer average vocal output levels across the two environments. Singers in the warehouse environment produced consistently higher vocal output than those in the piano room environment across all eight participants. The mean singing RMS level for the warehouse group was −20.94 dBFS (SD = 2.96), compared with −27.23 dBFS (SD = 4.13) for the piano room group, a difference of 6.29 dB. This difference was statistically significant (Mann–Whitney U=56.0 , p=0.0068 , one-tailed) with a large effect size ( r=0.617 ).

**Table 3:** Per-singer average vocal output levels across two acoustic environments.

Singer	Warehouse (dBFS)	Piano Room (dBFS)	Difference (dB)
S01	−19.75	−32.03	+12.29
S02	−15.50	−28.83	+13.33
S03	−26.65	−26.69	+0.04
S04	−19.80	−23.27	+3.47
S05	−21.55	−18.75	−2.79
S06	−19.75	−30.88	+11.14
S07	−22.01	−30.29	+8.28
S08	−22.49	−27.06	+4.57
Mean	**−20.94**	**−27.23**	**+6.29**
SD	**2.96**	**4.13**	

Values represent the mean of two recording takes per singer. Root-mean-square (RMS) levels are expressed in decibels relative to full scale (dBFS); SD denotes standard deviation.Bold values indicate the group mean and standard deviation.

**Figure 5:**
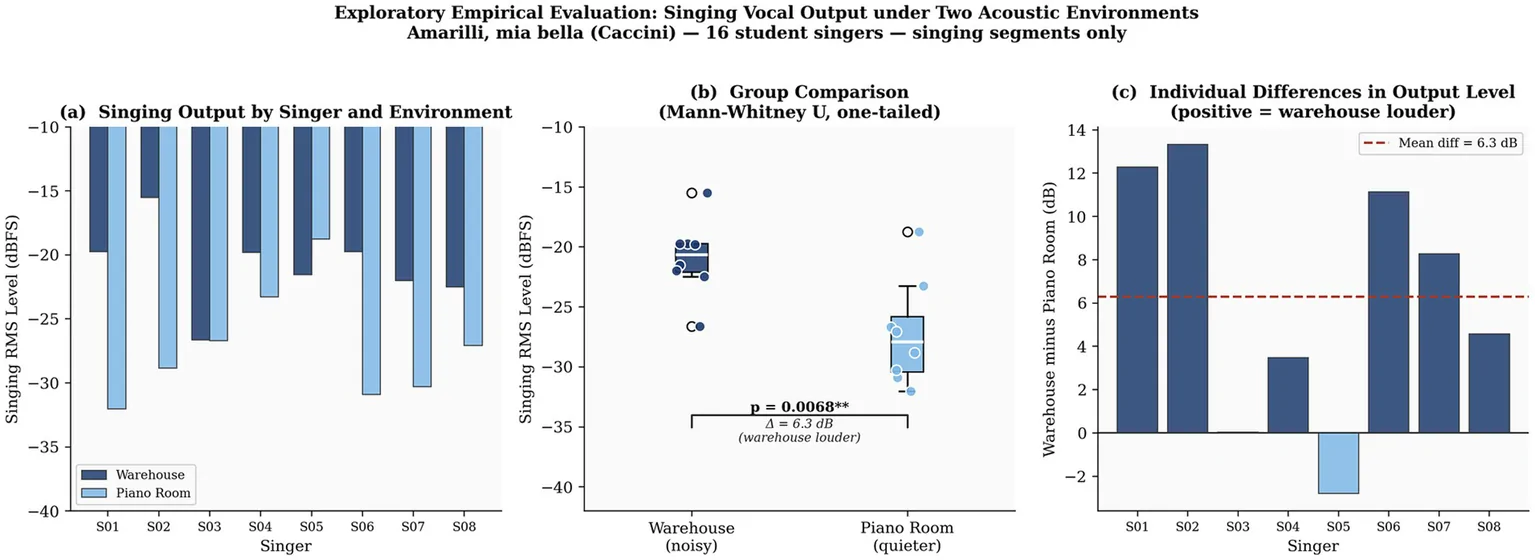
Exploratory empirical evaluation of singing vocal output across two acoustic environments (16 student singers, two independent groups, Amarilli, mia bella, Caccini; singing segments only). **(a)** Per-singer average root-mean-square (RMS) singing level for the warehouse (noisier) and piano room (quieter) environments. **(b)** Box-plot comparison with individual data points overlaid; asterisks indicate statistical significance (Mann–Whitney U = 56.0, *p* = 0.0068, one-tailed; *r* = 0.617). **(c)** Individual differences in singing output level (warehouse minus piano room, dB); positive values indicate higher output in the warehouse environment. All levels are expressed in decibels relative to full scale (dBFS). Results are directionally consistent with the Lombard-based prediction that singers increase vocal output in noisier environments.

Figure 5 presents the group comparison. Panel (a) shows per-singer output levels for both environments, demonstrating that the warehouse group was consistently louder than the piano room group across all eight singers. Panel (b) presents a box-plot comparison with individual data points overlaid, confirming the distributional difference. Panel (c) shows the relationship between estimated noise floor and total vocal output across both pooled environments; while the noise floor estimate from the first 5 sec of each recording was not a calibrated measure of ambient noise level and the group difference in noise floor did not reach significance (Mann–Whitney U = 41.0, *p* = 0.191), the positive association between noise floor and vocal output (*r* = 0.44, *p* = 0.012) is consistent with the direction predicted by the Lombard model.

These findings should be interpreted cautiously. RMS in dBFS is an uncalibrated, relative measure of level, and the two groups were recorded in different rooms; room acoustics and microphone placement, rather than vocal effort alone, may therefore contribute to the observed difference. The absence of a calibrated ambient noise measurement further prevents any direct estimate of the Lombard slope from this dataset. These constraints notwithstanding, the finding that singers in the noisier environment produced significantly higher vocal output, with a 6.29 dB mean difference and a large effect size, is directionally consistent with the Lombard-based prediction that vocal output rises in noisier environments; the design does not, however, support any quantitative inference about Lombard slope magnitude or formal validation of the framework.

## 5 Discussion

### 5.1 Why $P_{s}$ , not SNR alone, should anchor vocal health risk assessment

An indicator is only as useful as the quantity it measures, and SNR and $P_{s}$ do not measure the same quantity. SNR measures whether a vocalist can still hear themselves above the noise; $P_{s}$ measures what the body is doing to keep them there. Because these are different quantities, they need not move together, and the sensitivity analysis in Section 4.4 shows that under Lombard compensation they move apart. The reason they move apart is the point of this section, so it is worth stating carefully.

Consider a vocalist with a steep Lombard slope. As ambient noise rises, they raise SPL sharply to stay audible. The increased SPL widens the voice-to-noise margin, so SNR improves and SNR-based classification records lower risk. But that same SPL is produced by higher subglottal pressure, and higher pressure adds to phonotraumatic dose regardless of the resulting SNR (29). So the single act of compensating has two opposite readings: it lowers apparent SNR-based risk and raises real load on $P_{s}$ . The stronger the compensation, the wider this gap. This is why SNR alone can mislead: it does not merely undercount the load, it improves at the very moment the load grows and so reports the most effortful compensation as the safest state.

This failure mode most heavily affects the vocalists we know least about. The argument above scales with Lombard slope: the steeper the response, the larger the hidden load. Our parameters come from trained singers, who show relatively suppressed slopes (16) and lean more on proprioceptive than auditory feedback under masking (25). Untrained active vocal users, community choir members, street performers, and similar groups may compensate more strongly, in which case the concealment is larger for them than our professional-singer parameters imply. This is an extrapolation, not an observation of these groups, and testing it directly is a priority for future work.

Two limitations should be acknowledged. First, the observed divergence is directional rather than quantitative: across the tested slope range, mean $P_{s}\;$ changes by only approximately 0.2 ${\mathrm{cmH}}_{2}O$ . The analysis therefore shows that the two indicators can differ in sign, not that the resulting physiological effect is necessarily large. Second, whether such a difference accumulates into meaningful vocal fatigue under prolonged exposure remains an open question. Accordingly, our conclusions rest on the structural direction of the disagreement rather than its modest magnitude. None of this discredits SNR; it locates what SNR is for. SNR marks one thing $P_{s}$ cannot: the crossing into negative territory, where a self-reinforcing feedback loop begins and auditory self-regulation is lost. That crossing is a genuine change of state, and it is invisible to $P_{s}$ . So the two indicators answer different questions, one is communicative and the other physiological, and their disagreement is itself the signal. When SNR improves while $P_{s}$ rises, the vocalist is winning on audibility and losing on load at once, and an assessment reporting only the first has measured audibility, not vocal health.

This changes what a soundscape intervention must be shown to do. A pleasant masker can lift subjective quality, and with it SNR, without lowering the noise the vocalist actually competes against; on an SNR-only account this reads as protection, but the vocalist’s load is untouched. Whether an intervention protects active vocal users therefore cannot be read from a listener-centered metric alone; it requires asking separately what the intervention does to $P_{s}$ . Making that a question one can pose is what the dual-indicator approach adds.

### 5.2 Who bears the load, and what it asks of design

The concealment identified in Section 5.1 is not an abstract measurement problem; it falls on specific people in specific places. If SNR can improve while physiological load rises, then the populations most exposed to this discrepancy are those who sustain vocal output in noisy public spaces without the trained control that would moderate it, and who are least visible to a listener-centered assessment. These are not only professional voice users. Community choir members, outdoor music participants, tour guides, and street performers routinely phonate for extended periods in parks, squares, and roadside spaces, and they do so, in many cases, with the stronger Lombard responses reported for untrained voices (16), which is precisely the condition under which the concealment is largest.

That this population has remained invisible follows directly from how noise environments have been assessed. Environmental noise frameworks were built around the listener, the person exposed to noise, rather than the person who must produce sound within it. A listener-centered metric asks whether an environment is pleasant or intrusive to receive; it has no term for what that same environment demands of someone producing sustained voice inside it. The physiological burden on active vocal users is therefore not a burden these frameworks underestimate; it is one they are not structured to see at all. The present framework supplies the missing term, translating an ambient noise level into an estimate of the load it imposes on a voice competing with it.

This reframing has practical implications for spaces designed for sustained vocal activity, such as community plazas, park rehearsal areas, and outdoor performance venues, where listener-centered noise targets may not align with vocal-load requirements. An environment can satisfy conventional noise limits while still requiring active users to sustain elevated vocal effort for prolonged periods. Whether such spaces should incorporate vocal-load-aware acoustic criteria remains an open design question. In the meantime, lower-cost measures—including amplification, relocation, scheduling, and opportunities for vocal rest—may effectively reduce vocal demand. Because amplification is not always available or appropriate, the present framework is not intended to prescribe specific interventions, but rather to identify locations where vocal load is sufficiently elevated to warrant consideration of such measures. Where the framework can already offer an operational handle is in prioritizing and evaluating interventions. Because SNR marks the threshold at which auditory self-regulation is lost (Section 5.1), an intervention that keeps a vocalist’s estimated SNR above roughly +5 dB can be said to protect the communicative dimension of vocal health, even where its effect on absolute load remains to be quantified. More broadly, the framework provides an additional physiological perspective that complements conventional listener-centered assessments. Depending on context, interventions may include acoustic redesign, amplification, relocation, scheduling, or other practical measures. The framework therefore serves primarily as a diagnostic tool, complementing conventional Lden-based assessments rather than replacing them. By identifying locations where environmental noise translates into elevated vocal load for active users, it helps planners prioritize where interventions are likely to help most.

### 5.3 Limitations and future directions

Several limitations bound the claims made here. The *first* concerns the parameters. The Lombard slope and baseline SPL driving the analysis are drawn from trained singers recorded under stable laboratory accompaniment (16), whereas real urban noise fluctuates unpredictably and may provoke stronger compensation than those conditions capture. This makes the present estimates conservative in one direction, but not uniformly so: the subglottal-pressure estimator itself is calibrated for classically trained male phonation (Section 3.3). Styles using greater glottal adduction reach a given SPL at lower $P_{s}\;$ (22), so applying the estimator to higher-adduction or untrained voices, including several of the community groups for whom the argument in Section 5.2 matters most, would require style-specific recalibration. The populations of greatest interest are therefore those the current parameterization fits least well, and closing that gap is the most consequential extension of this work. A further limitation is what the estimates represent, even when the parameters fit. The framework estimates chronic vocal load, not clinical outcome, and establishing the relationship between estimated pressure and clinical voice outcomes will require longitudinal studies combining voice dosimetry with clinical follow-up.

A *second* limitation is what the framework leaves out of the voice itself. It estimates aerodynamic load but does not model the articulatory aspects of Lombard adaptation, including formant tuning, spectral tilt, and fundamental frequency shifts, each of which alters both vocal effort and how a voice projects against noise. A fuller model would couple the pressure estimate used here to these spectral changes rather than treating loudness in isolation.

A *third* limitation lies in the noise data. The city-scale analysis uses road traffic contours as the only source, while active vocal users in cities are also exposed to construction, crowd, and amplified-music noise that the EEA dataset reported under the END does not record. The maps therefore describe only one component of acoustic exposure, rather than total exposure; incorporating multiple sources would raise the estimated load and likely shift the risk geography. The three-country selection carries a related caution: it spans real variation in urban form and regulation, but cannot stand in for European soundscapes at large, and a wider set of agglomerations would test how far the present risk distributions generalize. The noise input itself, Lden, is an annual penalty-weighted average, so the framework characterizes chronic relative exposure rather than the acoustic conditions of any specific rehearsal or performance. Linking these estimates to instantaneous vocal behavior would require time-resolved noise data, which the END dataset does not provide.

Furthermore, because the framework relies on outdoor road-traffic noise data reported under the END, it is intended for outdoor urban vocal activity and does not extend to indoor occupational voice users such as teachers or call-center workers. The present framework assumes direct voice production under environmental noise and does not explicitly model communication assisted by amplification, hearing devices, or telecommunication systems. Extending the framework to these communication scenarios will be an important direction for future work. The present analysis maps where elevated vocal load is likely to occur. Quantifying the exposed population will require data beyond those reported under the END.

A *fourth* limitation is the exploratory evaluation in Section 3.6. Those recordings were made for teaching, not for a Lombard experiment: singers were not randomly assigned to the two rooms, ambient noise was not captured with a calibrated meter, and vocal output was measured as uncalibrated dBFS in two acoustically different spaces, so room acoustics cannot be cleanly separated from vocal effort. The result is consistent with the framework’s central behavioral assumption but does not test it; it is a directional check, not a validation. A purpose-built study, pairing wearable voice dosimetry with real-time noise dosimetry in the field, would both ground the load estimates ecologically and allow a direct test of whether the SNR– $P_{s}$ divergence of Section 4.4 appears in natural vocal behavior.

These limits qualify the framework without undoing what it establishes: that vocal health risk can be estimated at urban scale from open noise data on a physiologically interpretable basis, and that separating the communicative and physiological dimensions of that risk changes how an assessment should be read. What the framework currently offers is a defensible first estimate and a way of asking the right question; what it still needs is field data from the voices it is meant to protect.

## 6 Conclusion

In summary, this study extends urban soundscape assessment from the listener to the voice, introducing a framework for quantifying the physiological demands of communicating in noisy urban environments.

A framework for mapping vocal load. By integrating the Lombard effect, subglottal pressure estimation, and vocal dose theory, and by calibrating the approach against independent measurements from classical singers, we show that the physiological cost of urban noise can be estimated and mapped at city scale using publicly available noise data.

Audibility does not equal vocal health. Signal-to-noise ratio (SNR) measures whether a voice can be heard, whereas subglottal pressure ( $P_{s}$ ) reflects the effort required to produce it. Under Lombard compensation, these metrics can move in opposite directions: vocal compensation improves audibility while increasing physiological load. As a result, an environment may satisfy listener-centered acoustic criteria yet still place excessive demands on active voice users.

Toward a voice-aware soundscape paradigm. These findings complement the human-centered soundscape framework by incorporating the physiological burden on sound producers alongside the experience of listeners. Considering the voice, as well as the ear, provides a more complete basis for evaluating and designing healthy urban acoustic environments.

## Data Availability

The datasets presented in this study are available in online repositories. The names of the repository/repositories and accession number(s) can be found at: Open Science Framework (OSF): analysis code, https://doi.org/10.17605/OSF.IO/HUP2W, European Environment Agency (EEA) Spatial Data Infrastructure: road-traffic noise datasets, https://sdi.eea.europa.eu/data/853e72e4-4642-47d1-8556-2cc231bd43e0.
